# New Modeling Methods: Geographic Information Systems and Spatial Analysis

**Published:** 1997

**Authors:** William F. Wieczorek, Craig E. Hanson

**Affiliations:** William F. Wieczorek, Ph.D., is a research professor and director and Craig E. Hanson is a geographic information system analyst at the Center for Health and Social Research, Buffalo State College, Buffalo, New York

**Keywords:** geographical area, regional differences, environmental factors, context dynamics, alcoholic beverage sales outlet, location and density of outlets, AOD availability, drinking and driving, violence, crime, data analysis method, information source, computer technology, epidemiology, AOD prevention, scientific model, literature review

## Abstract

Geographic factors, such as the location of alcohol outlets or of neighborhoods with different socioeconomic status within an area, can influence the patterns of alcohol use and alcohol-related problems in that area. Geographic information systems (GIS)—computer-based systems to capture, store, retrieve, analyze, and display spatial data—are increasingly used to investigate the effects of such geographic factors. GIS offer several key capabilities that facilitate alcohol-related geographic analyses, including geocoding (the linking of descriptive data, such as driving-while-intoxicated [DWI] events, to a location on a map), informative visual displays, and calculation of distance and adjacency. Using GIS-based data, researchers can perform complex spatial analyses of alcohol-related behaviors and problems, such as determining the correlation between DWI rates and geographic locations. These types of analyses may help investigators to understand environmental influences on alcohol-related problems and to plan and target appropriate prevention and intervention approaches.

The biopsychosocial model of alcoholism posits that biological, psychological, and social factors jointly contribute to the development of alcoholism and alcohol-related problems. Although researchers have made significant progress in elucidating the biological and psychological elements of alcohol use and its associated problems, the analysis of the social component has substantially lagged behind. One approach to facilitating the complete integration of the social component into the biopsychosocial model is to examine geographic factors associated with alcohol use and alcohol-related problems.

Geographic analyses focus on the role of space (i.e., distance and area), place (i.e., space plus social and physical context), and location (i.e., the impact of being in specific spaces and places) in understanding social, behavioral, and natural phenomena, including alcoholism. Numerous findings of alcohol research have indicated that important geographic factors are associated with the use and abuse of alcohol. These geographic factors include spatial factors (e.g., the location of and distance between places where alcohol is sold [i.e., alcohol outlets]) as well as contextual and environmental factors that define specific places, such as local cultural characteristics and socioeconomic status. Furthermore, the results of analyses of these factors can be summarized in attractive visual displays, such as maps. Until recently, however, such geographic approaches to alcohol epidemiology were rarely implemented because of substantial technical difficulties, such as the labor required to place a large data set onto a map and insufficient computer software for performing these spatial analyses.

The development of geographic information systems (GIS) will allow researchers to greatly increase their understanding of geographic factors that influence alcohol use and its associated problems. The resulting new modeling methods, which are based on spatial conceptualizations, permit the improved integration of geographic and social factors into alcohol research and are comparable in significance to the advanced perspectives on biological processes that have been provided by new internal imaging technologies. Thus, spatial modeling approaches can generate new research questions as well as provide answers to these and existing questions.

The simplest definition characterizes GIS as “automated systems for the capture, storage, retrieval, analysis, and display of spatial data” (Clarke et al. 1996, p. 85). Martin (1996) has provided another definition of GIS, which is based on the terms making up the acronym. Thus, “geographic” indicates that GIS analyze the spatial relationship of objects (e.g., points, polygons, lines), using a coordinate system (i.e., a system in which the location of each object defined by its location on an x axis and a y axis) and scale (i.e., the ratio of the distance displayed by the GIS to the actual distance on the ground). The term “information” refers to the fact that GIS represent collections of data that provide information about the spatial location of the variable under investigation as well as characteristics (i.e., descriptors) associated with that location. Finally, the term “system” implies that GIS provide an organized approach to managing and querying these data. For investigating specific health-related and social applications, however, these characteristics of GIS must be combined with expertise in a specific field of research, such as alcohol studies. This article introduces geographic concepts in alcohol research, reviews current GIS, and presents some spatial models.

## Association of Alcohol Use and its Consequences with Geographic Factors

Geographic factors can affect alcohol use on different scales (e.g., international, regional, and local) and at different levels of intensity. Some of the most readily noted and accepted geographic influences on drinking manifest themselves on an international, cross-cultural scale. For example, MacAndrew and Edgerton (1969) have documented many examples of cross-cultural differences in drunken behavior, which can range from egregious behavior to quiet introspection. Similarly, significant differences exist in beverage preference and total alcohol consumption between wine-drinking countries (e.g., France and Italy) and other countries. Cultural influences on drinking persist even after a person emigrates to another country, where he or she may interact with new, local influences. The resulting drinking practices then reflect both sets of influences (Kitano et al. 1988).

Geographic patterns of alcohol consumption and its consequences also exist in the United States. These regional differences result from historical immigration patterns and the interaction of immigrants with their new local environments. Thus, drinking practices vary most prominently between “wetter” regions, mainly in the Northeast and Midwest, and “drier” regions in the South. Compared with drier regions, wetter areas are characterized by lower rates of alcohol abstention and greater alcohol availability. Complex relationships exist, however, between alcohol availability and alcohol-related problems. For example, the consumption per drinker and level of adverse drinking consequences are higher in the dry areas than in the wet regions (Hilton 1988). This observation indicates that more research is needed to better define and understand the factors associated with these regional patterns.

Geographic influences on drinking and the associated problems also are apparent on an even smaller scale, as in communities and neighborhoods. For example, one study of small cities in southern California found that local differences in alcohol availability significantly predicted differences in violence and crashes (Scribner et al. 1994, 1995). Using sophisticated geographic modeling techniques, Gruenewald and colleagues (1996*a*) showed that alcohol availability associated with specific types of outlets (e.g., restaurants) significantly predicted the incidence of single-vehicle nighttime crashes, which are most likely to involve alcohol. Similar contributions of geography and community context also have been recognized in other areas of epidemiology (Aangeenbrug et al. 1997). These findings indicate that by linking individuals and their environment, researchers can rigorously examine key epidemiologic concepts.

## GIS as an Emerging Technology in Alcohol-Related Epidemiology

The usefulness of geographic analyses in addressing epidemiologic problems has been recognized since the 19th century, when John Snow, by mapping all cases of a cholera outbreak in a London neighborhood, identified a specific water pump as the source of the outbreak. Nevertheless, GIS can be considered an emerging technology, because only recently have improvements in computer capabilities and decreases in their costs made sophisticated spatial analysis feasible for the study of health and social problems. In fact, some observers have considered the increasing use of GIS to be one of 1995’s 10 most notable developments in epidemiology (*Epidemiology Monitor* 1996). In alcohol research, technologies such as GIS extend the biopsychosocial conceptualization of and contribute to our knowledge of regional, spatial, and contextual factors in alcohol use and its associated problems. Moreover, the knowledge derived through GIS may influence the design and implementation of prevention and treatment programs.

The integration of GIS into health and social research has been made possible by major advances in computer technology, software, and data availability. The most important advance was the development of a national, digital base map for the United States. A digital base map is a computer file containing the x and y coordinates (i.e., map coordinate data) of all objects of interest. This national base map, which is known as “topologically integrated geographic encoding and referencing” (TIGER), was developed by the Census Bureau (Marx 1990). It contains the address ranges for every block and street of every community in the United States. In fact, TIGER files provide street-level data that often include thousands of street segments for a geographic area the size of a single county. This scale and accuracy provide epidemiologists with the digital infrastructure for GIS-based health and social research, including alcohol research.

### Capabilities of GIS

To understand the role of GIS-based measures in alcohol research, it is essential to understand the capabilities of GIS and how they can facilitate spatial analysis by producing maps, other visual images, quantitative measures of spatial phenomena, and models (Wieczorek in press). The key capabilities of GIS that facilitate geographic analysis include geocoding, informative visual displays, and calculation of distance and adjacency.

#### Geocoding

An essential feature of any GIS is the ability to link descriptive data, such as the occurrence of a driving-while-intoxicated (DWI) event, to a geographic reference (e.g., a building or street block) that has specific x and y coordinates on a map. This process is called geocoding, or address matching, because the GIS will match the “address” of the descriptive event with a database of addresses that already are associated with geographic references. This geocoding process forms the basis of the GIS’s ability to display, analyze, and model information.

GIS geocoding is most often performed by automatically matching the descriptive data and the TIGER location data. For example, geocoding allows epidemiologists to determine how many DWI events have occurred on a certain street block in a certain community. However, geocoding is not limited to point or address data. For example, TIGER boundary files—databases of census and political boundaries—include geographic units, such as census areas (e.g., tracts), cities, counties, and States. Thus, ecological data of interest (e.g., mortality rates) can be matched to such geographic areas. The availability of TIGER has significantly improved the feasibility of successful geocoding, although some limitations may result from insufficient information from rural areas, the construction of new housing developments, and idiosyncratic addresses in some regions.

GIS also allow easy data access and management. Thus, researchers can formulate specific queries (e.g., to identify the locations of bars and the frequency of DWI events in a community) to examine specific data elements. The data then can be used in further analyses (e.g., how bar locations affect the frequency of DWI events).

#### Informative Visual Displays

Because visual images are a particularly powerful method of conveying information, the ability of GIS to display data or the results of complex analyses represents another key capability of this technology. The adage that a picture is worth a thousand words may be an understatement when it comes to conveying the information contained on maps. GIS-based maps can be examined as images on a computer monitor or as detailed hardcopy maps, allowing their dissemination in various formats, including video map files, Internet postings, and printed materials, that can be used for numerous purposes. This flexibility is enhanced further by the availability of a wide range of tools of map design (i.e., cartography), such as color, symbols, shading, and scale, to highlight relevant information.

GIS-generated maps can provide a simple geographic inventory (e.g., indicate the location of all DWI events that occurred within a certain time period in one community) or present the results of more complex analyses. Furthermore, GIS data can be reclassified quickly to display different spatial patterns. For example, the locations of alcohol-related crashes may vary by the time of day—late-night crash locations may differ greatly from midday crash locations. This type of data analysis and display is easily accomplished using a GIS, but would be time consuming and limited if done by hand.

**Figure 1:**
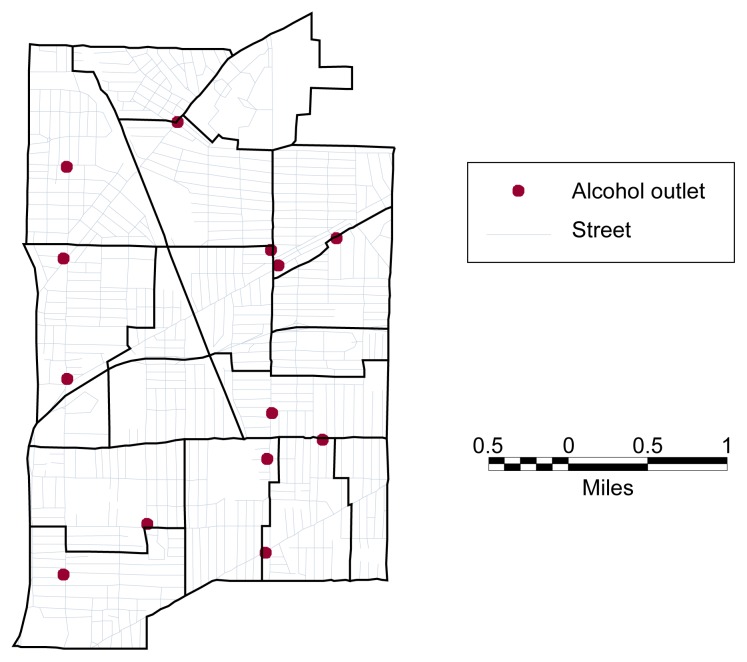
Illustration of the overlay functions of geographic information systems. The map displays three different variables, or layers of information, for a hypothetical area: the boundaries of census tracts in that area, the

Another important display feature of GIS-based maps is their overlay function, or their ability to examine or combine multiple variables (also called coverages or layers of information) for the same geographic area. For example, GIS-based maps can simultaneously display the locations of census tracts, streets, and alcohol outlets within a community (see figure 1). Furthermore, the overlay function also facilitates the clustering, or aggregation, of point data into geographic areas. For example, the number of alcohol outlets for each census tract of a community can be determined, thereby providing a measure of alcohol availability at the tract level. By linking the analyst’s data (e.g., the locations of single vehicle crashes) to meaningful variables, such as population or transportation characteristics, aggregation facilitates the calculation of rates of certain events. For example, one can calculate the rates of single vehicle crashes per 10,000 persons or of crashes per 1,000 miles of road using GIS-based data.

#### Calculation of Distance and Adjacency

**Figure 2:**
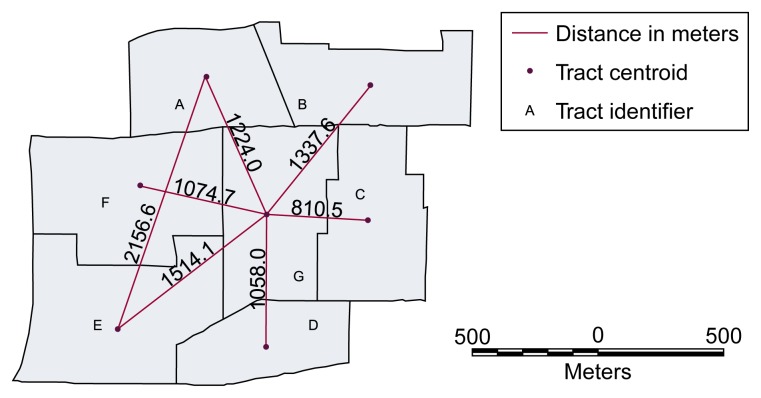
Illustration of the distance and adjacency functions of geographic information systems (GIS). The map of a hypothetical area shows the boundaries of seven tracts within that area. GIS can calculate the distance between the center points (i.e., centroids) of these tracts as well as determine which tracts are adjacent to each other (e.g., tract A is adjacent to tracts B, F, and G).

Because location may be a potent predictor of alcohol-related problems, it is essential to calculate the spatial relationships between individual spatial objects (e.g., bars and census tracts) when developing quantitative geographic models. Accordingly, another important feature of GIS is their ability to calculate the distance between spatial objects as well as adjacency of areas. Using simple geometry, GIS can determine the distance between points; centroids of polygons (i.e., the center points of areas such as census tracts); and network, or street, pathways (see figure 2). In addition, GIS can identify areas that are adjacent to one another (see figure 2). One use of the distance function is to create a spatial variable by selecting and counting the number of events within a certain distance of a specific location. For example, using a GIS database one could calculate the number of assaults within 100 feet of each bar as a measure of bar-related violence.

GIS can employ different approaches to calculate the adjacency and/or distance measures required for spatial analyses. Some analyses (e.g., analyses of the general potential, which is described later in this article) use a distance measure for spatial relationships. Other models may use a binary (i.e., yes or no) measure of whether areas are adjacent, or they may use the length of the shared boundary between areas as an adjacency measure. Most GIS can calculate these spatial relations. To perform statistical analyses or develop models based on these data, specialized modeling software may be needed with more complex spatial statistics. As with other statistical problems, several approaches may be suitable for any given modeling problem, of which the researcher must choose the most appropriate one.

## GIS and Spatial Analysis

Spatial statistics are statistical techniques for measuring and modeling how phenomena are related geographically. An important characteristic of spatial analyses is the existence of spatial dependence (also called autocorrelation) in many data. Spatial dependence means that the value of a variable is a function of its location (Berry 1997). For example, household income generally does not differ randomly across various neighborhoods of a community. Instead, people are more likely to have incomes similar to people in their own neighborhood than to people in more distant neighborhoods.

**Figure 3:**
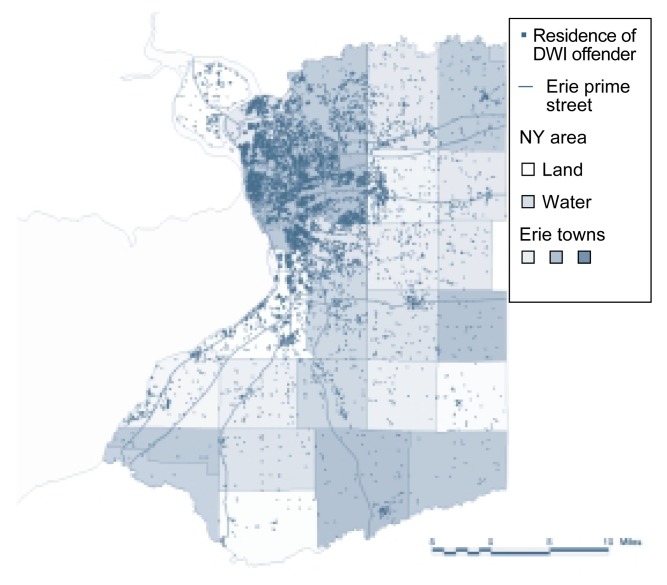
Geographic information system-based map displaying the residences of 15,551 driving-while-intoxicated (DWI) offenders in Erie County, New York.

**Figure 4:**
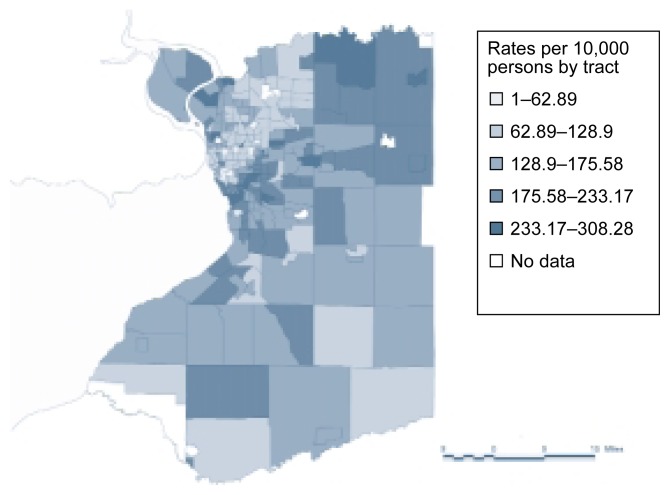
Geographic information system-based map displaying the driving-while-intoxicated (DWI) rates per 10,000 persons in the census tracts of Erie County, New York. The map shows that patterns and substantial differences in DWI rates exist among the census tracts.

One example of the integration of GIS-based data and spatial analysis is a study of DWI offenders in Erie County, New York (Wieczorek and Coyle 1998). This analysis used census tracts and the addresses of 15,551 DWI offenders (see figure 3) to calculate DWI rates for each census tract. Visual inspection of the resulting map (see figure 4) clearly indicates that substantial geographic differences in the DWI rate exist among census tracts. This information can then be used to target prevention efforts or to identify correlates of DWI.

One method to determine whether spatial dependence exists for DWI rates, based on data such as those described in the previous paragraph, is to calculate a variable that represents the correlation between DWI rates and geographic location. One such variable is known as a “general potential” (Ricketts et al. 1994, p. 68). The general potential derived for each tract is based on the DWI rates in various census tracts and the distances between those tracts. Thus, tracts that are located close to high-DWI-rate tracts are assigned a high value for general potential. An analysis by Wieczorek and Coyle (1998) found that a positive correlation existed between the general potential and the actual DWI rate for each tract: Tracts that were close to high-DWI-rate tracts and therefore had a high general potential were likely to have high DWI rates themselves. These findings indicate that spatial dependence exists for DWI rates, because tracts located close to each other tend to have similar DWI rates. They also show that DWI patterns are not random and that area effects can extend beyond the tract boundaries.

Another commonly used and statistically powerful test for general spatial dependence is the determination of the so-called Moran coefficient (Garson and Biggs 1992). The Moran coefficient usually ranges in value from −1 to 1, with values close to zero indicating spatial randomness (i.e., no spatial dependence). A positive Moran coefficient indicates positive spatial dependence—that is, similar values tend to be located close to one another. Negative values of the Moran coefficient, which are relatively uncommon, indicate negative spatial dependence (i.e., unlike values tend to be located close to one another).

Gruenewald and colleagues (1996*a*,*b*) have pioneered the development of software and multivariate models (i.e., models that include more than one variable) that use spatial dependence in alcohol research. These models have included multiple levels of data (e.g., individual data, such as answers from individual survey respondents, as well as environmental data, such as alcohol availability) and coefficients of spatial dependence for each variable. This research has found, for example, that significant spatial dependence exists among such factors as type of alcohol outlet (e.g., restaurant or liquor store) and single-vehicle crashes. Such analyses may lead to new environmental approaches to prevention, such as limitations on the number of specific types of alcohol outlets in a geographic area or the targeting of DWI enforcement measures to certain locations.

### Contour Maps

One problem with the interpretation and modeling of spatial data using the methods described in the previous section is that these analyses assume that the variable under investigation is uniform within the geographic units studied (e.g., that the rate of DWI events is the same in all areas of a census tract). This uniformity may not exist in the real world, however. For example, the rate of DWI events may differ between main thoroughfares and residential side streets or among residential areas. One method to account for this variability is to present the data as a continuous process across the area studied using isoline, or contour, maps, which connect points of equal value. For instance, on topographical or weather contour maps the lines connect points of equal elevation or temperature. Similarly, contour maps can be generated that display points of equal rates of DWI events. Contour maps to date have rarely been used in alcohol studies, largely as a result of the lack of previous geographic analysis in the field and the difficulty of creating contour maps without the use of a GIS.

The generation of contour maps generally requires the availability of numerous measurement points to use as base points for the contours. Thus, when trying to create a contour map of a larger area (e.g., a county), the number of tract centroids may not be sufficient to create a continuous surface. To overcome this problem, one can overlay a grid of cells of a certain size on the entire county. Using a geostatistical technique known as kriging, one can then use a limited number of points (e.g., the tract centroids) to create a model that provides a value for each of the grid cells. Kriging is a method of deriving a value for an unsampled point (i.e., any point other than the centroids) based on the values of the centroids surrounding that point (Isaaks and Srivastava 1989). The method was originally developed to locate mineral ore based on limited sample data. To date, kriging has rarely been used in health and social research. The technique has great potential, however, for contributing significantly to analyses such as models of the geographic distribution of health problems and their associated consequences in society.

**Figure 5:**
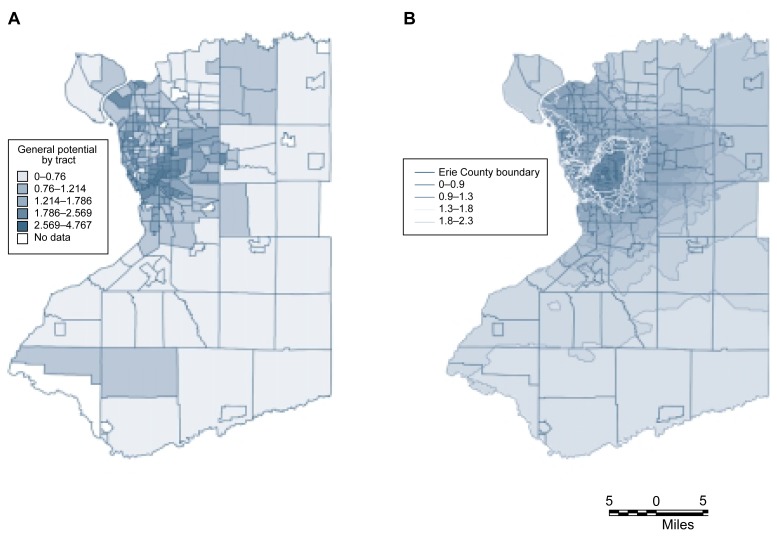
Geographic information system-based maps of the general potential^1^ for driving-while-intoxicated (DWI) rates in Erie County, New York. (A) The map illustrates the general potential by census tract. (B) The general potential is displayed on a contour map, which was generated by overlaying the entire county with a grid having a cell size of 1,000 feet. Using statistical analyses, the general potential for each grid cell was determined, and points of equal general potential were connected by contours. This analysis better displays trends in general potential than does the tract-based map. ^1^The general potential is a measure for determining whether spatial dependence exists for certain variables (i.e., whether the value of the variable is a function of its location). For example, tracts that are located adjacent to high-DWI-rate tracts are assigned a higher general potential than tracts located adjacent to low-DWI-rate tracts.

**Figure 6:**
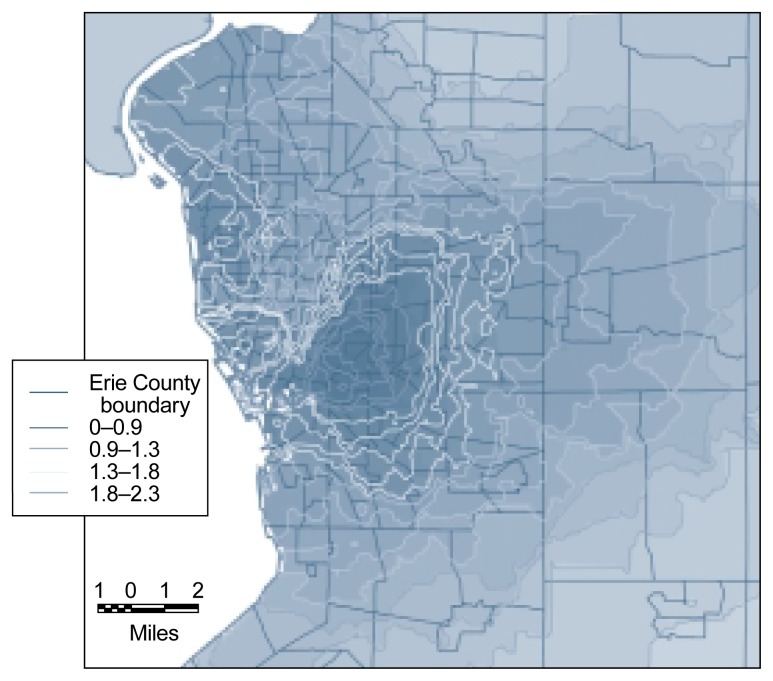
Geographic information system-based contour map of the general potential^1^ for driving-while-intoxicated (DWI) rates in Buffalo, New York. (This map is a more detailed section of map B shown in figure 5.) The figure illustrates that not only the city itself but also the outer suburbs have a high general potential. ^1^The general potential is a measure for determining whether spatial dependence exists for certain variables (i.e., whether the value of the variable is a function of its location). For example, tracts that are located adjacent to high-DWI-rate tracts are assigned a higher general potential than tracts located adjacent to low-DWI-rate tracts.

The advantages of contour maps over tract-based spatial analyses can best be illustrated with an example, such as the distribution of the general potential for DWI events across Erie County, New York. A tract-based display identifies regional differences between census tracts in general potential, but these distinctions appear rather crude (see figure 5, left panel). For a more detailed analysis, the county was overlaid with a grid having a cell size of 1,000 feet, and the general potential for each grid cell was determined using the general potential values of the tract centroids and the kriging technique. The centroids of each grid cell then were used to develop a contour map (see figure 5, right panel). This contour approach allows more sophisticated analyses than the tract-based approach, because it displays spatial trends of the general potential in greater detail and represents differences in general potential as a continuous process over space. Thus, the contour map identifies one main peak of high general potential, a secondary peak to the northwest of the main peak, and a general east-west trend in general potential. The more detailed contour map of the city of Buffalo and its surrounding suburbs (see figure 6) illustrates that high DWI rates in the city also may affect suburbs that border the city and even outer suburbs, because these areas also have a high general potential. In the future, similar types of analyses may be used to estimate the spatial distribution of actual alcohol use and alcohol-related problems.

## Other Spatial Analytic Techniques and Models

The general potential, kriging, and contour maps are just a few examples, of the geographic modeling techniques facilitated by GIS. Numerous other spatial methods and models exist whose detailed description would exceed the scope of this article. For more extensive information on GIS, geographic analyses in health services research, and spatial data analysis and statistics, the reader is referred to several recent reviews (Garson and Biggs 1992; Ricketts et al. 1994; Cressie 1993; Bailey and Gatrell 1995; Fotheringham and Rogerson 1995).

### Applications of Spatial Dependence

Slight variations in the interpretation of spatial dependence can provide additional insight into the concept of geographic relationships (e.g., Griffith 1993). These different interpretations can be used for developing a variety of models. Regardless of the specific interpretation and model used, however, the most salient feature of spatial dependence is that it violates a basic assumption underlying classical, nonspatial, statistical models. These classical statistical models (e.g., regression analyses) cannot account for spatial effects on the variable under investigation; rather, they assume that the variable is independent of its location. As a result, using these models for spatial analyses can lead to incorrect results in tests of statistical significance. Even advanced nonspatial statistical techniques, which allow for the analysis of various levels of measures, cannot fully overcome this limitation, because they assume spatial homogeneity. Therefore, a major contribution of GIS-based geographic models is that they can account for spatial dependence, thereby allowing for valid tests of statistical significance, especially in multivariate models.

### Local Indicators of Spatial Association (LISA) Techniques

The analysis of global measures of spatial dependence has some limitations. For example, it does not identify local variations in the geographic distribution of the variable studied. These local areas are of interest, because specific places may have a negative or positive spatial dependence that differs from the spatial dependence of the entire area studied. Although such areas can be identified using contour maps, this approach does not provide a statistical test of significance of the spatial dependence for each area. Statistical techniques known as local indicators of spatial association (LISA) can identify statistically significant spatial dependence in these smaller areas (Anselin 1995). Although LISA techniques currently are limited to univariate analyses (i.e., they can analyze only one variable at a time), they can provide a more detailed assessment of spatial dependence compared with more traditional techniques. In particular, LISA techniques can more readily identify negative spatial dependence. Thus, Wieczorek and Hanson (1997) have used LISA methods to identify counties with positive or negative spatial dependence in alcohol-related mortality. This method may be particularly useful for identifying places with specific types of alcohol-related problems.

### Spatial Cluster Analysis

As described previously in this article, spatial dependence analyses determine whether the occurrence of a certain phenomenon is dependent on location. Spatial cluster analysis is another approach to determining whether a phenomenon is spatially random. This technique is particularly appropriate for point data (e.g., the occurrence of DWI events, where each event is considered a “point”). A spatial cluster exists when the number of points that are geographically close to each other is greater than expected. Techniques for spatial cluster analysis usually also allow significance testing and may be used to investigate clustering in both space and time (Ricketts et al. 1994). Numerous opportunities exist for the use of spatial clustering methods in alcohol research. For example, this technique could be used to examine spatial and temporal factors that influence alcohol-related crashes or alcohol-related violence. Spatial cluster analysis also may be used to identify “natural” communities or neighborhoods—that is, communities or neighborhoods that are similar based on drinking patterns or socio-demographic characteristics rather than political or census boundaries.

### Noncomputerized Data Analysis

Although computers have greatly facilitated the analysis of complex data, the usefulness of a “human” visual approach to analyzing geographic data should not be underestimated. Just as classical data analysts should examine nongeographic data through visual analyses, such as scatter plots and distributions, spatial data analysts must use their abilities by examining visual displays of their data, typically through maps. The ability of the investigator to interpret information presented on a map may allow the identification of complex patterns and potential relationships that can lead to new research questions.

## Summary and Conclusions

GIS and methods of spatial analysis are coming of age for applications to a broad variety of health and social research issues, including alcohol studies. The initial use of these methods by alcohol researchers indicates a strong potential for major contributions to our conceptualization and understanding of alcohol use and its related problems. GIS-based approaches provide powerful methods of exploring data to generate new hypotheses as well as techniques to test these hypotheses.

The potential for applying GIS in alcohol research is virtually unlimited. For example, the identification of spatial associations provides a powerful rationale for future research into the processes that account for these findings. Thus, a geographic association between specific types of alcohol outlets and crime would suggest the need for research analyzing the activities associated with the specific forms of alcohol availability that lead to crime at these locations. Drinking and driving and alcohol availability are two areas that are particularly appropriate for GIS-based analysis. GIS-based approaches also can improve studies of cultural differences in alcohol use, whether on a neighborhood or regional scale, because these approaches allow culture to be defined by integrating individual and environmental data. Furthermore, specific geographic studies of the multiple contexts in which alcohol is consumed are important to understanding environmental influences on alcohol problems. Finally, the planning and targeting of alcohol-related health services, such as prevention and treatment, have intrinsic geographic components.

GIS also can be used to create spatial variables based on a person’s specific location and can be combined with other individual-level data. For example, for each person in a survey study, the number of alcohol outlets within a specific distance of the person’s home can be calculated as an individual measure of alcohol availability. In addition, GIS can play an important role in analyses to determine if certain health and social processes are scale dependent. This information would be essential to understanding social and contextual influences on alcohol-related problems. To maximize the contribution that GIS and spatial analysis can make to alcohol research, investigators must understand the technology and use creativity in its application.
